# Potential Natural Fiber Polymeric Nanobiocomposites: A Review

**DOI:** 10.3390/polym12051072

**Published:** 2020-05-07

**Authors:** K. M. Faridul Hasan, Péter György Horváth, Tibor Alpár

**Affiliations:** Simonyi Károly Faculty of Engineering, University of Sopron, Sopron, 9400 Gyor, Hungary; horvath.peter.gyorgy@uni-sopron.hu

**Keywords:** biofiber, nanofiller, biocomposites, nanobiocomposites, polymer, functionality, reinforcements

## Abstract

Composite materials reinforced with biofibers and nanomaterials are becoming considerably popular, especially for their light weight, strength, exceptional stiffness, flexural rigidity, damping property, longevity, corrosion, biodegradability, antibacterial, and fire-resistant properties. Beside the traditional thermoplastic and thermosetting polymers, nanoparticles are also receiving attention in terms of their potential to improve the functionality and mechanical performances of biocomposites. These remarkable characteristics have made nanobiocomposite materials convenient to apply in aerospace, mechanical, construction, automotive, marine, medical, packaging, and furniture industries, through providing environmental sustainability. Nanoparticles (TiO_2_, carbon nanotube, rGO, ZnO, and SiO_2_) are easily compatible with other ingredients (matrix polymer and biofibers) and can thus form nanobiocomposites. Nanobiocomposites are exhibiting a higher market volume with the expansion of new technology and green approaches for utilizing biofibers. The performances of nanobiocomposites depend on the manufacturing processes, types of biofibers used, and the matrix polymer (resin). An overview of different natural fibers (vegetable/plants), nanomaterials, biocomposites, nanobiocomposites, and manufacturing methods are discussed in the context of potential application in this review.

## 1. Introduction

Manufacturing industries are turning to increasingly sustainable, environmentally friendly economic production with the rapid improvements in science and technology. Nanobiocomposite (NBC) materials have emerged with potential features and performances for a variety of applications in different sectors (aeronautical, automotive, furniture, packaging, transportation, medical, and defense sectors), as alternatives to conventional materials. NBC materials are formed with two, three, or more components; one is in matrix form and the others are in particle or biofiber forms. Whenever any load is applied to NBC materials, it is shared equivalently with every part. The greatest benefits of polymer-based NBC materials are the higher productivity on an industrial scale, the ease of processing technology, and a reduction in the manufacturing costs. Fibers, especially biofiber-reinforced biocomposites (BCs) and NBCs, offer better advantages compared to conventional composites [1,2,3,4]. 

**Figure 1:**
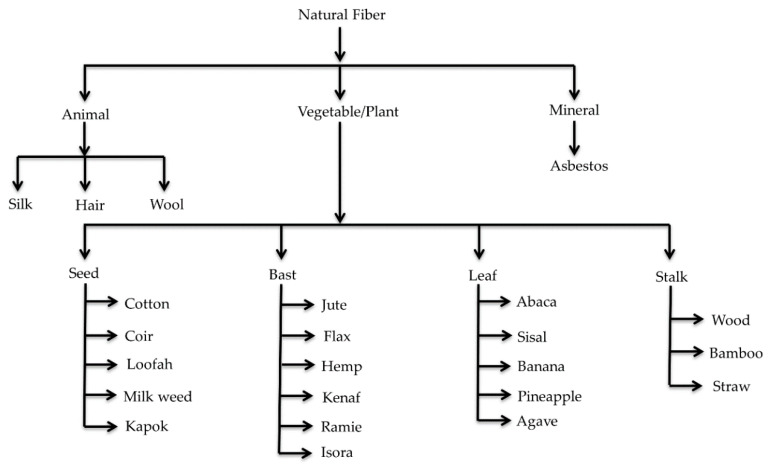
Natural fiber classification, according to the origin, with examples. Adapted with permission from Elsevier [9]. Copyright Elsevier, 2019.

The combination of organic natural fibers (Figure 1) and inorganic or organic polymers and nanoparticles has a high potential for improving mechanical performances, and thus expanding the areas of application. Recently, various inorganic nanoparticles were studied for incorporating them with biofibers in the matrix to form NBCs especially for their biodegradability. This results in the development of an interfacial bond between the biofibers and polymers in a composite system, whereas the organic phase helps to form an inorganic matrix [5,6,7,8].

The longer the polymeric chain of the fiber, the better its compatibility for use in BC materials; in particular, cellulosic fibers have this property. They have hydrogen bonds on the adjacent rings and phenyl rings on the backbone. Polymeric fibers also have some limitations, such as a low stiffness, which is responsible for providing low protection against heat. 

When the composites are made from petroleum-based fibers, greenhouse gases are generated, whereas cellulosic fiber-based composites could minimize this challenge [10,11]. Natural fibers exhibit very good insulation properties, which is why they can be used in the construction and automotive sector [12,13]. There are few technologies available to produce short, long, and continuous fiber reinforcements, among which continuous fibers are compounded with thermoplastic and another one by thermosetting polymerization. Both short and long fiber polymer composites have been applied in the automotive industry. 

The structure of thermoplastics can be crystalline, amorphous, or even semi crystalline, and is affected by the different processing technologies of the polymers. Thermoplastic polymers are made by different methods, such as injection molding, extrusion, and compression molding. Different natural fibers are used for reinforcements, along with the matrix, to enhance the strength and performance of the composites. Often, compatible additives are also added to enhance the performance of composites [14,15,16]. 

Recently, nanoparticles have become more popular for enhancing the mechanical and functional performances (flame retardancy, antibacterial, and anticorrosion, etc.) in BCs. Nanofillers are replacing traditional microscale filler materials. The homogeneous and uniform distribution of nanoparticles in the BCs accelerates the mechanical, thermal, and molecular movability [17]. In the case of NBC production, the larger aspect ratio of nanofillers provides better reinforcements, which is why researchers are becoming more and more interested in such material. The characteristics of NBCs are highly influenced by the nanofillers’ specific surface area [18]. Nanofillers are available in either inorganic or organic forms. Titanium dioxide (TiO_2_), zinc oxide (ZnO), silicon dioxide (SiO_2_), and polyhedral oligomeric silsesquioxane (POSS) nanoparticles are inorganic in nature. On the other hand, carbon nanotube, graphene oxide (GO), and montmorillonite nanoclay are organic nanomaterials [19,20]. Researchers have been trying to develop hybrid composites by combining biofiber/biofiber and biofiber/artificial fibers with polypropylene, poly lactic acid, polyester, polyurethane, poly vinyl ester, and so on. 

However, the most compelling feature of an NBC is that it exhibits a higher strength, even though it does not inherently possess the same stiffness. Numerous NBCs reinforced with cellulosic fibers have been reported by researchers [21,22,23,24]. Nano-based reinforced polymeric composites are also generating dramatic revolutions in this sector for sophisticated applications, especially to enhance flame retardancy, water repellency, corrosion, and antibacterial properties. Nanomaterials could be applied as filler materials, along with biofibers and matrix polymer, in composites for acquiring better performances [25]. 

**Figure 2:**
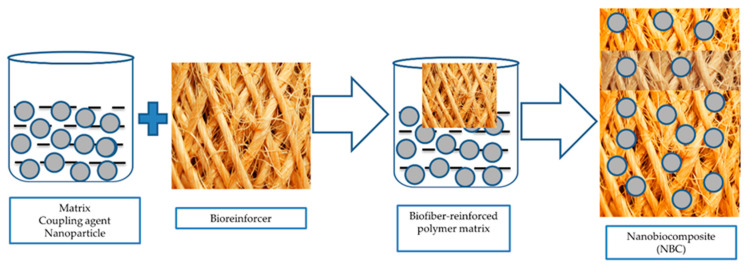
Formation mechanism of nanobiocomposites (NBCs).

BCs have largely been used in the construction sector, especially for the development of building materials with a superior flexibility and improved physical, mechanical, and functional properties. The nanodimensional phase has a remarkable effect on NBC properties, especially the thermal, electrical, optical, mechanical, catalytic, and electrochemical properties. The outstanding performance characteristics of BCs and NBCs have made them superior over conventional composites, as illustrated in Table 1. The presence of nanomaterials significantly affects the degree of thermoset curing, formation chemistry, movability of the polymer chain, and the order for the polymer chain and crystallinity in the composite matrix [26]. A polymeric graphene nanocomposite was developed by the solution mixing technique to increase the elastic modulus by 24%, which would be significant in the automotive sector [27,28]. Nanomaterials, fillers, and additives could address some basic and major issues for the revolutionary development of NBCs in the near future. A potential formation mechanism of NBC is illustrated in Figure 2. Therefore, the need to understand and study various NBCs and their formation and functional perspective is important. Considering this, we shall discuss and review the different reinforcements of natural fibers achieved by applying suitable methods, technology, and materials. 

## 2. Natural Fibers

**Figure 3:**
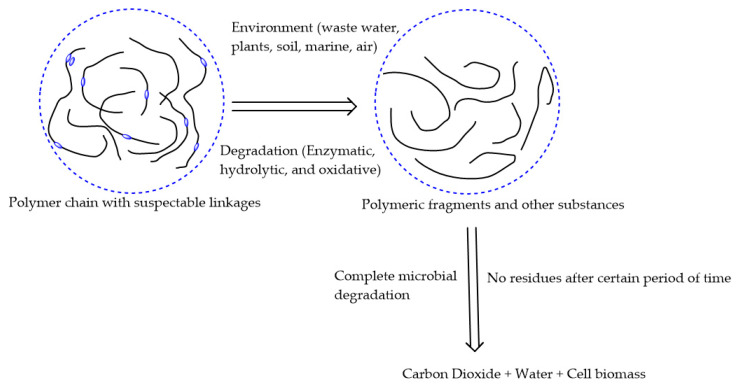
Biodegradation of biofiber-based composites. Adapted with permission from Reference [8]. Copyright Polymedia Publisher GmbH, 2009.

**Figure 4:**
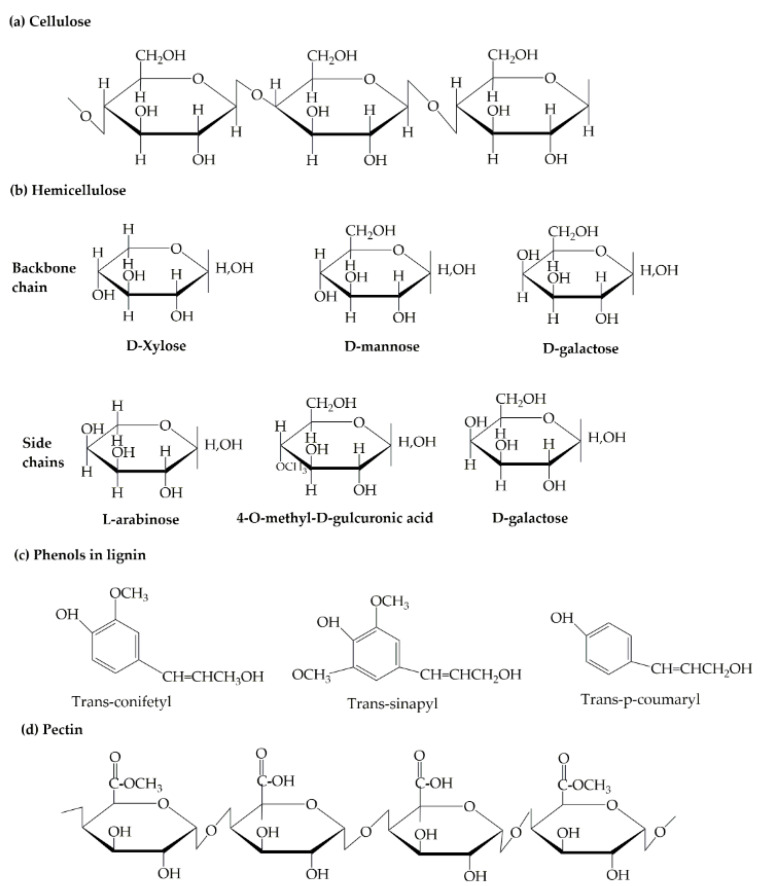
Different chemical structures of natural fibers: (**a**) Cellulose; (**b**) Hemicellulose; (**c**) Phenols in lignin; and (**d**) Pectin. Reproduced with permission from Elsevier [10]. Copyright Elsevier, 2015.

Natural fibers are extensively available in nature, and are found all over the world. Natural fiber-based composites are becoming popular day-by-day and replacing synthetic fiber-oriented composites due to their outstanding biodegradability (Figure 3), renewability, decomposability, stiffness, higher length to weight ratio, and low cost ($0.25–$4.25/kg) [29,30]. Natural fibers are categorized into four main classes: seed fibers (cotton, coir, and kapok), leaf fibers (sisal, agave, pineapple, and abaca), bast fibers (kenaf, ramie, hemp, jute, and flax), and stalk fibers (wood, straw, and bamboo) [31]. The extensively used natural reinforcers are cotton stalk, bamboo, rice straw, kenaf, hemp, abaca, and flax fibers [32]. The chemical composition (Figure 4) of these biofibers significantly influences the performance of composites, so we have studied their chemical properties, which are provided in Table 2 [33,34,35]. 

Cellulose is the main chemical component of all plant-based natural fibers. It is the most noteworthy organic component produced by plants that is ample in the environment. Cellulose is composed of a long chain of glucose polymer units that are connected to form microfibrils. 

Hemp is one of the most rapidly growing natural plants and is extensively used for building materials and textile fibers [36,37]. Hemp fibers are biodegradable, abundantly available in nature, and renewable. BCs made from hemp fibers have been used in automotive panels for a long time as eco-friendly, economical, and sustainable products. Nanomaterials have an important role for enhancing the strength of cellulosic fibers. Additionally, nanoparticles can enhance the mechanical properties of NBCs through their incorporation with cellulosic fibers (such as hemp), inorganic additives, and tributyl citrate plasticizer. Research on NBCs produced by reinforcing hemp fiber and SiO_2_ with polylactic acid (PLA) was conducted to investigate the thermomechanical performances (storage modulus of 19.93 GPa). The efficient and proper dispersion of silica nanoparticles resulted in enhanced mechanical properties.

Coir fiber is produced by the outer part of fruits in coconut. Coir is used to produce diverse environment-friendly and biodegradable products for commercial, industrial, and household applications. It has widespread usage in mats, geotextiles, sacking, garden articles, and automotives. In terms of minimizing cost, coir could be an ideal choice for replacing the glass fiber to produce thermoplastic reinforcement composites, due to its outstanding mechanical properties. The higher content percentage of coir (60%) increases the tensile (by 35%) and flexural (by 26%) strengths in coir/polypropylene reinforcements [42]. However, the increased fiber content (coir) negatively influences the internal bonding strength and water resistance [42]. Coir fiber has very good capability with regards to resistance against moisture, salty water, and heat. Therefore, coir could be a potential candidate for NBC production in the future.

Flax fibers are normally collected from the stem, which is a few times stronger than that of cotton [43]. Flax is a good substitute for synthetic fibers to produce NBCs. Flax fibers have superior mechanical properties in comparison to glass fibers. Moreover, the density of flax is also nearly half comparing to glass fibers. Therefore, when composites are formed with flax fibers, they exhibit a lighter weight and higher strength than synthetic materials [44]. Flax fiber composites made of thermoplastics, thermosets, and biodegradable materials display amiable mechanical performances [45]. Flax fiber is able to be reinforced with graphene nanoparticles for producing low-cost and feasible NBCs through multifaceted processing routes. Therefore, an increased flame retardancy, along with tensile strength (61% higher than of the case without nanofiller), was reported for graphene reinforced with flax/epoxy composites [35]. 

Cotton is a widely used cellulosic fiber throughout the world. When cotton is reinforced with PLA to form BCs, it exhibits good mechanical characteristics, with significant improvements in the tensile strength and Young’s modulus (as shown in Table 3), without any reduction in deformation at breaking point [46]. Cotton burs could be a potential replacement/alternative in blending composites, rather than other agricultural plant fibers that enhance the thickness, swelling, and water absorption, for post-thermal treatments [47]. Cotton has the potential to be used as a suitable reinforcement for producing low-cost composites. A good thermal stability of the BC was reported to improve the adhesion between cotton and the polymer matrix through curtailing access of the oxidizing gas on the interface [48].

Ramie is a green functional bast fiber with a silky appearance, higher absorbency, air permeability, and lower wrinkle characteristics. The gummy materials obtained from ramie fibers need to be removed through a degumming process before effective industrial processing can take place [49]. It is one of the strongest biodegradable, natural fibers, that exhibits a high strength, and antibacterial and flame retardant properties [50,51]. PLA is brittle in nature, which limits the application of PLA-based polymers, but the reinforcement of natural fiber (such as ramie)/nanofiller could reduce this effect and improve the thermal and mechanical properties. The surface pretreatment of fiber is also becoming poplar for significantly enhancing the mechanical properties of biocomposites. In this regard, ramie fiber was treated with silane−1 that exhibited good tensile (59.3 ± 1.2 MPa), impact (18 KJ/m^2^), and flexural (135 MPa) strengths on PLA/ramie composites [52].

**Figure 5:**
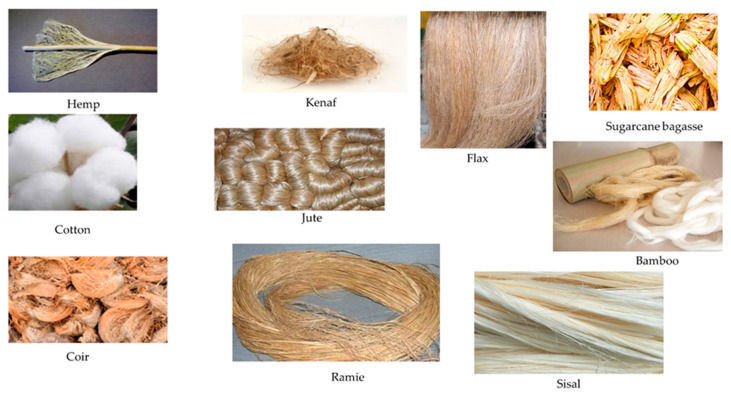
Images of natural biofibers [34,59,60,61,62,63,64,65,66,67,68].

Sisal has a good stiffness, durability, and resistance to salty water, which is why it has been applied for a long time in twines, ropes, papers, filters, mattresses, and carpets. Some exceptional advantages of using sisal (Figure 5) fibers are related to its (1) lower density, (2) nonabrasive nature, (3) lower cost, (4) lower energy consumption, (5) higher possibility of filling level, (6) biodegradability, (7) higher specific properties, and (8) generation of an agricultural-based economy in rural areas [58].

Agave is another potential natural fiber which is receiving attention from researchers and manufacturers due to its reproducibility, lighter weight, and economical aspects. Even though raw agave fibers could play a significant role in improving the reinforcement properties, some pretreatment processes of fibers have also been reported to enhance better effects [69,70]. The elastic modulus of composites made from agave fibers increases with a higher loading percentage of fiber content; a similar trend was also observed for the yield strength in terms of agave/high-density polyethylene (HDPE) and agave/Polypropylene (PP) composites [71]. The manufacturing method, materials for matrix, and application of some widely used biopolymers are shown in Table 4.

Polymers are composed of many repeating subunits, such as monomers, that are chemically bonded. They are mainly classified into two categories: one is natural polymers (like cellulose, pectin, protein, lignin, and hemicellulose) and the other is synthetic polymers (e.g., poly ethylene terephthalate (PET), nylon, PP, PS, and LDPE). There are also other types of modified natural polymers, such as viscose. BCs are currently receiving a tremendous attention because of their biocompatibility and biodegradability, making them potential replacements for petroleum-based plastic materials [94]. 

**Figure 6:**
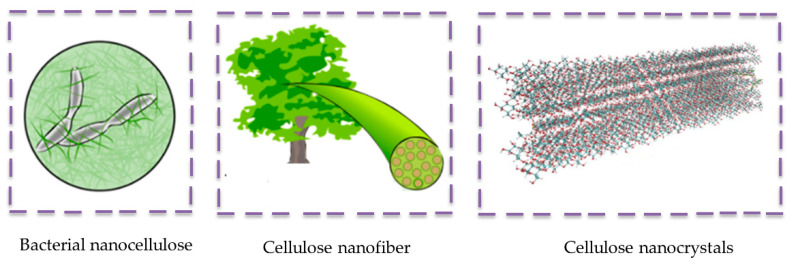
Images of bacterial nanocellulose, a cellulose nanofiber, and cellulose nanocrystals. Adapted with permission from reference [98,99]. Copyright MDPI, 2020 (BNC and CNF). Copyright NAS (National Academy of Sciences of United States of America), 2018 (CNC).

Nanocelluose is a general term used for cellulose nanocrystals (CNC), cellulose nanofibers (CNF), and bacterial nanocellulose (BNC), as shown in Figure 6. Natural fibers (ramie, jute, sugarcane bagasse, and coconut, etc.), wood, herbs, and organisms can be used for the isolation of cellulose nanocrystals. However, the removal of all non-cellulosic parts (hemicellulose, lignin, pectin, wax, and other extractives) is necessary before the isolation process can start. The cellulosic contents are exposed by different pre-treatment processes (bleaching, mercerization, pulping, and enzyme) of lignocellulosic fibers. Natural nanoparticles can be synthesized by using various methods, such as mechanical processes, including sonication, grinding, and homogenizing, or chemical treatments (ionic liquid and acid hydrolysis), or by using both types of combined processes (steam explosion) [95,96,97]. CNF entails amorphous regions which are composed of stretched cellulosic nanofibers providing higher mechanical properties, whereas CNC has a higher rigidity, resulting in an elevated Young’s modulus, tensile strength, and thermal stability [98]. On the other hand, BNCs have a higher molecular weight and are synthesized from different bacterial species (Rhizobium, Sarcina, Agrobacterium, and Pseudomonas), providing higher water retention and Young’s modulus values. Nanocellulose could contribute to obtaining a higher tensile strength, higher viscosity, higher elastic modulus, and lighter weight in NBCs. However, there are also some challenges facing cellulose nanoparticle incorporation and proper dispersion in the polymeric matrix [97].

## 3. Polymer/Matrix

A matrix holds all of the reinforcing fibers and agents together in a composite to transfer/share any external stress within the constituents for providing protection against any degradative processes, either in a mechanical (impact damage, delamination, high temperature, creep, and water absorption) or chemical form. The matrix is also termed a base material, and plays a critical role in composites carrying tensile loads in the structure [100,101]. There are four important matrix types: (1) metallic, (2) polymeric, (3) carbon, and (4) ceramic. The most used matrixes in manufacturing companies are polymeric resins, which are mainly thermosetting polymers and thermoplastics. Thermoset matrixes are crosslinked during the curing process. The crosslink is produced upon heating or by adding the curing agents. Consequently, thermoset plastics become stronger and stiffer, which has made them an attractive polymer matrix in traditional fiber-reinforced composites, such as carbon or glass fiber-based composites. Polyester resin, epoxy resin, poly amide, novolac, polyurethane, urea formaldehyde, melamine resin, and vinyl esters are popular thermoset polymers. Extrusion molding, reactive injection molding, spin casting, and compression molding methods are used to produce thermoset polymers [102,103]. 

Conversely, thermoplastic polymers are made from plastic polymers that require a convenient temperature (Table 5) for processing and retain a solid phase after cooling. The molecular weights of thermoplastics are very high, and the polymeric chains are interconnected through intermolecular forces. The prime advantage of this polymer is that it can be reheated again, without any major changes in the original properties for any kind of reformation. PP, PLA, LDPE, PS, HDPE, PVC, acrylonitrile butadiene styrene (ABS), and Teflon are some common examples of thermoplastic polymers. There are various processing techniques available to provide specific shapes for thermoplastic polymers, such as calendaring, and extrusion, injection, and compression molding [104,105]. 

## 4. Surface Treatment of Biofibers before Composite Formation

The surface of natural fibers can be modified by means of physical, chemical, and mechanical processes. Physical methods are more sustainable and assist in reducing the polar difference between the matrix and fiber surface, whereas chemical processes are used to reduce the degradation against moisture absorption [30]. In the case of the physical approach, the chemical structures are not changed, but the adhesion property between the biofiber and the matrix is improved through enhancing the interfacial adhesion [106]. On the other hand, extensive studies have been reported for chemical treatment methods of biofibers, such as BC production [107,108]. The compatibility of biofibers can be increased by using surface pretreatments, which reduce the dependency on synthetic fiber-based composites. 

**Figure 7:**
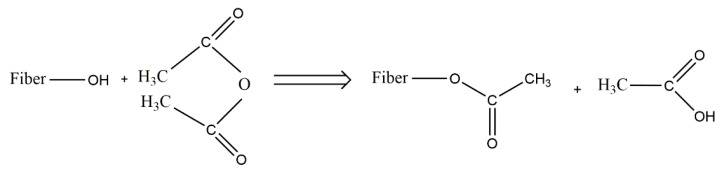
Reaction mechanism of a biofiber and maleic anhydride. Adapted from Elsevier [111]. Copyright Elsevier, 2012.

The interaction of the natural fiber matrix is enhanced through chemical modification by means of maleated polymers and maleic anhydride. The hydrophilic characteristics of natural fiber are reduced through the strong interaction between the hydroxyl group of lignocellulosic fibers and maleic anhydride, as shown in Figure 7. A covalent or hydrogen bond is formed when the maleic acid is grafted with PP and the natural fiber surface (hydroxyl group). The mechanical (tensile, impact, and flexural) strengths of BCs are improved when maleic acid is used to graft the polymers for facilitating bonding with bio fibers in the matrix [109]. The functional, mechanical, and color properties of BCs are deteriorated when exposed to heat, sunlight, humid environments, and radiation for the formation of gas molecules, moisture absorption, and changes in the polymeric structure. Such kinds of challenges can be eliminated by treating biofibers with appropriate chemical additives or matrix compatibilization [30]. Silane is another prominent method of treating fibers containing different functional groups through interacting with both the hydrophilic and hydrophobic ends to form a bridge in the matrix [110].

In the case of mercerization, an alkaline treatment is performed to remove the excess lignin, oil, wax, and impurities from the exterior surface of natural fibers. The short fibers are exposed and the depolymerization of cellulose is performed in this stage. The hydroxyl group is ionized to alkoxide when the alkaline solution is added to the natural fibers [111], as shown in Equation (1).

Fiber – OH + NaOH → Fiber – O – Na + H_2_O (1)

## 5. Preparation of BCs and NBCs

Certain influential parameters need to be considered before BC and NBC production, including the types of biofiber, moisture content of the biofiber, temperature, pressure, type of required performance, and volume fraction of biofibers. Additionally, the length, chemical composition, and aspect ratio of natural fibers also have significant effects on the manufacturing and performance of composites [112]. However, the deformability of composites decreases with an increase in fiber volume. Natural fibers can be processed up to 200 °C for producing NBCs without fiber degradation [113]. A temperature exceeding this range may result in poor performances due to changes in the physical, chemical, and mechanical properties through oxidation, depolymerization, recrystallization, decarboxylation, dehydration, and hydrolysis [114]. Compression molding, injection molding, extrusion molding, resin transfer molding, and sheet molding are used for BC production, whilst compression molding is the most popular and widely used technology [115]. In the case of compression molding, preheated fiber materials are compressed with a high pressure until solidification occurs. The pressure, temperature, heating time, and cooling time are some of the most important parameters that need to be considered for compression molding [11,116]. Sheet molding is one form of the most popular compression molding methods for manufacturing composite panels. Extrusion molding is another, being the easiest technology with which to make composites with higher strengths and stiffnesses. In this method of processing, the thermoplastic polymers are stored in the hopper as granulates and molted by heat in the barrel, which is finally cooled after obtaining the desired shape [112]. Injection molding is used for the mass production of composites, where polymeric granules are put into the hopper and then molted, before being injected into the chamber and placed in a mold, where they solidify after cooling down [112,117].

**Figure 8:**
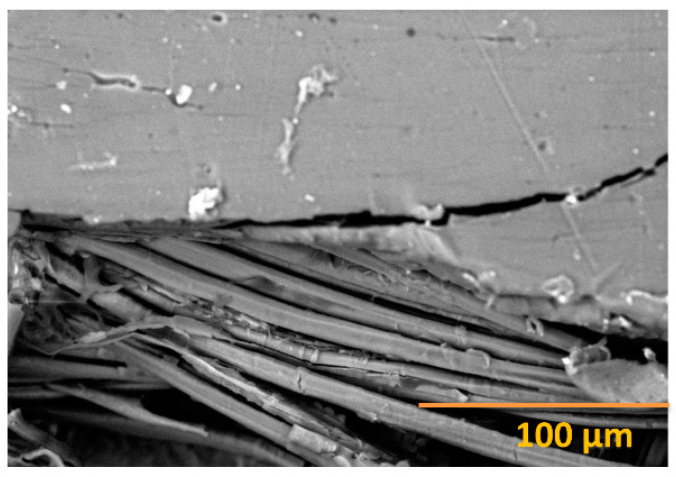
Tensile test for the PLA/flax fiber composites breaking area after the test. Figure republished from Alpár, Markó, and Koroknai (2017), with permission from John Wiley & Sons [123]. Copyright John Wiley & Sons, 2017.

BCs are made of a polymeric matrix (resin) or nanomaterials (recent studies) with natural fibers for reinforcement. These are made of organic or inorganic compounds that are natural or synthetic. The materials used are structured through mimicking living constituents during the processing that are hardened and strengthened by the matrix, but need to ensure biocompatibility [118,119,120]. Both renewable and nonrenewable polymeric ingredients can be utilized for the formation of the BC matrix. This matrix holds the fibers together, which enhances the strength; thus, it achieves good protection capabilities against mechanical deformation and environmental degradation, as well as through transferring the load uniformly to the whole surface area. Plant-based fibers (jute, flax, hemp, cotton, coir, agave, and ramie), cellulosic papers, various byproducts, and wood are abundantly available biofibers in nature. Natural BCs have a relatively lighter weight, higher stiffness, and higher strength to width ratio. BCs have widespread applications in the aerospace, automotive, packaging, medical, and construction sectors [121,122]. A prominent surface interaction result was previously reported for flax and PLA-based composites [123] in terms of the tensile strength by researchers in our lab, as shown in Figure 8, where both continuous and short fibers were reinforced with thermoplastic polymer (PLA). BCs are advantageous because they have so many unique characteristics, such as sustainability, renewability, recyclability, biodegradability, a flexible design, better productivity, a smaller carbon footprint, and low costs.

## 6. Natural Filler Reinforced Polymeric NBC

**Figure 9:**
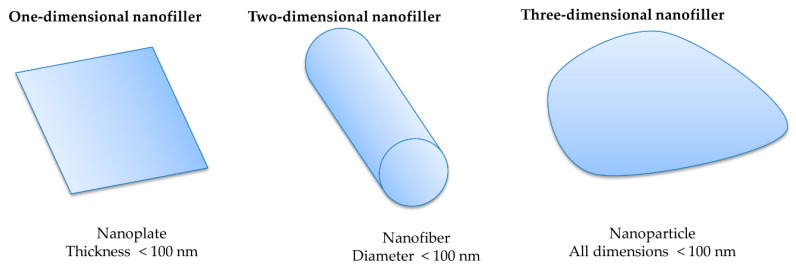
Nano objects used for nanobiocomposites, according to ISO/TS27687 (2008). Adapted from reference [135]. Copyright IntechOpen, 2011.

Fillers are important parts of composites that contribute in particle, fragment, fiber, sheet, and whisker forms, either as natural or artificial materials, as shown in Figure 9. Some lignocellulosic fibers have been utilized as filling materials for the last 3000 years as a reinforcement ingredient, along with other polymeric constituents [124,125]. Recently, nanofillers have been considered to be highly potential components for enhancing the polymeric properties and mechanical performances of NBCs. Currently, cheaper, lighter, stronger, and thinner composites are a target of researchers and manufacturers, who hope to achieve such nanofillers in superior material selection [126,127]. When the size of a larger surface polymeric matrix is shrunken to a smaller area in the nm (nanomaterial) range, various flexible functionalities appear, along with an excellent mechanical strength (tensile strength and stiffness), compared to the raw form or without nano-treated composites [128]. One of the key benefits of nanoparticle-incorporated BCs is that they can enhance the behavior at a high temperature, without changing the processing conditions and melting temperature [129]. Thermoset polymers become brittle when undergoing crystallization. These challenges can be eliminated by incorporating biofibers and nanofillers (TiO_2_, SiO_2_, carbon nanotube, ZnO, and graphene oxides) [130,131]. The grafting of nanofiller also increases the density of composites, which results in a hardness in NBCs. Besides, natural fibers have better specific properties than synthetic fibers, which, in combination with another reinforcing agent (nanofiller), enhances the performances of NBCs.

**Figure 10:**
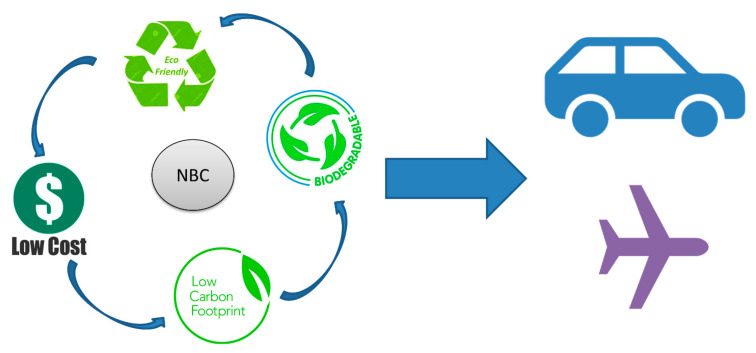
Sustainable features and potentiality of NBCs.

When nanoparticles are distributed in the BC matrix for specific functionalization purposes, NBCs are produced. The size of the nanomaterials used in NBCs is usually less than 100 nm. NBCs exhibit better performances compared to traditional BCs. In recent times, extensive studies have been conducted for applying different nanoparticles as convenient nanofillers in NBCs [132,133,134] through providing environmental sustainability, as illustrated in Figure 10. Polymeric NBCs (thermosets, thermoplastics, and vitromers) can easily be reinforced with biofibers and matrix [37,135]. Researchers have developed various processing techniques using different polymeric ingredients in the matrixes through reinforcement with different clays for achieving functional properties.

## 7. Nanoparticle-Based BCs (NBCs) 

With the fast development of nanoscience and technology in various fields, nanomaterials have shown their significant potential importance. When the dimension of a minimum of one reinforcer in a polymeric composite is in the range of 1 to 100 nm, the material is termed as NBC. Recently, green NBCs have also been receiving attention due to their renewability and biodegradability. Nanomaterials have very good mechanical, optical, and thermal characteristics that can be easily implemented as nanofillers for functionalizing various NBCs [136]. Nanomaterials can be formed naturally, as biproducts of suitable reactions, by applying various methods, or be mechanized for specific functionalities, which may result in different physico-chemical characteristics [137,138]. There are various types of nanomaterials (NMs), such as graphene oxide, silver, TiO_2_, ZnO, SiO_2_, and polyhedral oligomeric silsesquioxane (POSS)), which are used for diverse potential applications. However, for NBC production, TiO_2_, SiO_2_, carbon nanotube, ZnO, and graphene oxides are receiving more attention and are still being reported by different scientists [105,139,140]. Biofibers are naturally hydrophilic, but epoxy resins are hydrophobic, which results in weaker bonding in the NBC matrix, providing poor mechanical properties. Researchers have reported several methods (such as plasma treatment and alkali treatment) for overcoming this challenge, but the reinforcement of nanofillers on the NBC is still providing better potentiality compared to others [141,142]. Biofibers associated with NBC formation also do not always comply with the expected mechanical performances, thermal stability, and barrier resistance, but the incorporation of nanofillers in the NBC could eliminate such drawbacks [143]. The synthesized nanoparticulates do have some drawbacks, in addition to many other advantages, so researchers are increasingly becoming involved to find more potential solutions. We have discussed some commonly used nanoparticles corresponding to different NBC developments and prospects in this review. 

### 7.1. TiO_2_-Based NBC

Researchers are becoming more interested in TiO_2_ NPs around the globe, in line with the advancement of composite materials. Nano TiO_2_ has a very good compatibility with thermosetting epoxy resin, in addition to its corrosion resistance, chemically inert, low-cost, and nontoxic characteristics [144]. Dip coating is a popular method for applying nano TiO_2_ on composite-forming substrates to improve the biocompatibility [145]. A biodegradable natural NBC was reported for packaging materials, where PLA was used for dispersion purposes, PP was employed as the matrix, and TiO_2_ was used as the nanofiller. The melt-blending process was applied to convert the materials into biodegradable composites, which showed both UV resistance and water repellent properties [146]. The crystallization enthalpy measurement equation is shown in Equation (2). The crystallization enthalpy for a 100% crystalline polymer sample was reported to be 93.0 g/j for PLA and 201.1 g/j for PP and the mass% filler was a compatibilizer and nano−TiO_2_ [147,148].

(2) $$X_{c}=\frac{\Delta H}{\Delta H_{m}^{o}\cdot \left[1-\frac{\mathrm{mass}\%\cdot \mathrm{filler}}{100}\right]}\times 100$$

where *X*_c_ is the degree of crystallinity, ∆*H* (g/j) is the crystallization enthalpy, and Δ$H_{m}^{o}$(g/j) indicates the melting enthalpy. The samples need to be heated for 5 min at 200 °C for assessing the isothermal crystallization. The temperature is elevated from 25 to 200 °C with the gradient of 10 °C/min. Then, the temperature is cooled down quickly (−50 °C/min) to the required temperature (110, 105, 100, 95, and 90 °C) for crystallization assessment. Following this, crystallization is allowed for 120 min at an isothermal temperature. The samples need to be reheated again at 200 °C with the same temperature gradient for observing the melting characteristics [148]. The incorporation of TiO_2_ can partially eliminate the reduction of crystallinity in TiO_2_/PLA nanocomposites [149].

The effect of TiO_2_ NP was studied for the mechanical, thermal, and water absorption features of natural flax fiber that was reinforced with epoxy resin composites. The epoxy resin was modified with different percentages of nano TiO_2_, and was 50 nm in size. The tensile strength increased by 10.95%, impact strength by 20.05%, and flexural strength by 10.45%. The water diffusion was also reduced to 31.66% with the addition of TiO_2_ [150]. The interfacial bond between the biofiber and polymeric matrix plays a significant role in improving the mechanical properties in the composite. Natural fiber-based composites have a very weak interfacial bonding strength and thermal stability, which limits their applications in load bearings. The incorporation of TiO_2_ NM can enhance the affinity between the fiber and matrix, which facilitates an increase in the mechanical and functional performances.

### 7.2. Silica-Based NBC

Silicon dioxide is another prominent nanofiller for reinforcing natural fibers in polymeric NBCs. The bubbles and agglomeration of silica nanofiller can reduce the mechanical performances of the NBC. However, the uniform distribution of fillers in polymeric BCs and abolition of bubbles prior to curing in the matrix can help produce an NBC with an optimal performance [151,152]. The breaking point of BC is another major challenge which can be enhanced by using silica nanoparticles. Besides the thermal, optical, and mechanical properties of the NBC, good interfacial bonding can also be significantly improved between the jute fiber and matrix by using silica nanoparticles [153]. SiO_2_ nanomaterials have drawn the attention of present researchers due to their non-toxicity, photo bleaching, surface functionalizing, and other properties, which can be obtained through different physical and chemical modifications [154,155]. The thermal performance of biofiber-based composites is not satisfactory, but the grafting of SiO_2_ in the matrix could play a significant role in enhancing the flame retardancy. A successful NBC was reported to enhance the bonding strength between the epoxy resin and ramie fiber by using three different nano-silica (1% each), along with an increase in the mechanical strength (10.87% to 20.06% increase in tensile strength, 20.5% to 32.88 % increase in flexural strength, and 14.78% to 32.49% increase in modulus of elasticity) and thermal performances [8]. A hybrid composite with increased thermal stability, stiffness, impact strength, and creep resistance properties was reported, achieved by incorporating SiO_2_ with flax fiber and PP/HDPE composites [156].

### 7.3. Graphene-Based NBC

Graphene-based NBCs are also drawing the attention of researchers, especially in the field of construction (for producing suitable building blocks) and medical sectors, for developing environment-friendly biomimetic approaches through using in situ grafting, host-guest interactions, noncovalent bonding, and polymerization reactions of free radicals [157]. Their higher Young’s modulus, electrical conductivity, and tensile strength have made them a promising nanomaterial for functionalizing bioinspired fibers for aerospace, car, and aeronautical fields. Only a small portion of graphene could significantly contribute to enhancing the functional and morphological properties of BCs. It was also noted that only an addition of 0.5 wt % of chemically converted graphene (CCG) could enhance the Young’s modulus by 170% and tensile strength by 70% [158]. In another study, a lighter weight NBC was reported to have increased mechanical, morphological, and thermal characteristics, and could be applied in automotive, building, and aeronautical sectors through incorporating graphene nanoparticles with biofibers (*Hibiscus cannabinus*) [159]. The main drawback of using a high concentration of graphene oxide in NBC is the filler to filler agglomeration, so it is recommended that an optimum loading of nanoparticles is employed [159]. However, graphene-based NBC processing is a little challenging and thus may provide poor performances in some cases [160]. The graphene provides strength in the composites through covalent bond formations with a reduced toughness, but it could be tuned through controlling the chain of molecules in the polymer [161]. 

### 7.4. Carbon Nanotube-Based NBC

A carbon nanotube (CNT) is considered an excellent nanomaterial for modifying the natural fiber surface through increasing the compatibility between the biofiber and polymer in the matrix. Besides, the polymeric materials used in BC materials should have an excellent resistance against the damage from any mechanical deformation, thermal instability, and chemical change to be suitable for space or automotive industries; in this regard, CNT could be an ideal selection, requiring very little loading as a prominent nanofiller [162,163,164]. CNT is a very good candidate for enhancing the interfacial mechanical strength in the NBC, due to its superior thermal and mechanical properties [165]. A promising reinforcing property was reported for a CNT network on a jute fiber surface in an NBC through a hierarchical structure [166]. The addition of CNT in a polymeric composite increases the damping property through creating interfacial slipping, along with associated friction. An enhancement of the damping property of 6% was proposed for multilayered NBC through modifying flax with CNT in other research [167].

### 7.5. ZnO-Based NBC

Zinc oxide nanoparticles have been extensively studied for their numerous applications in diversified fields. This NM has high potential for biomaterials, medical applications, wastewater treatment, and the electronics sector. A study was conducted concerning the ZnO nanomaterial biocompatibility, antimicrobial performance, and multiphase morphology of biomimetic nanocomposite materials with ZnO/sodium alginate/hydroxyapatite-oriented granules and ZnO/hydroxyapatite hydrogels. This study demonstrated the Zn^+^ behavior of different composite materials [168]. The formation reaction of ZnO was shown by the authors as follows:

(3) $${\mathrm{Zn}}^{2+}+2{\mathrm{OH}}^{-}\to 2{\mathrm{OH}}_{4}^{2-}\;\mathrm{and}\;\mathrm{Zn}{\left(\mathrm{OH}\right)}_{4}^{2-}\to \mathrm{ZnO}+2H_{2}O+2{\mathrm{HO}}^{-}$$

The average particle size of ZnO was shown to range within 12 to 30 nm in a previous study. The distribution of the nanoparticle was homogeneous, along with a very good compatibility in terms of the thermal property. It was also reported that the sol-gel method was suitable for synthesizing NMs with a size of less than 50 nm [169]. A bioplastic film was blended with ZnO NPs, synthesized through a solution casting method by using chloroform as a solvent [94]. It was also shown that low concentrated ZnO NPs could be applied for packaging materials. The focus on temporary biodegradable implants is growing steadily, especially in medical sectors. ZnO could be incorporated with BCs for producing potential flame-retardant and ultraviolet-protective NBCs [170].

## 8. Application of BCs and NBCs

**Figure 11:**
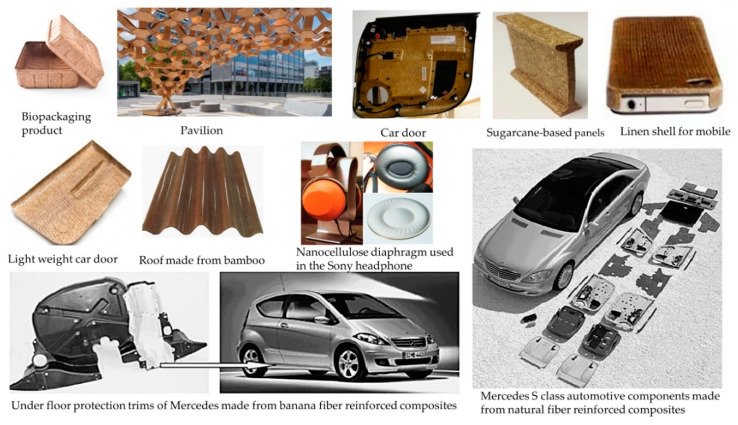
Different applications of BCs and NBCs. Under floor protection trims and S class of Mercedes are produced with the permission from Elsevier, 2008; Nanocellulose diaphragm (2011) was reprinted with the permission from author [171,172,173,174,175,176,177,178,179].

NBCs formed by polymeric matrix reinforcement with natural fibers have widespread applications in different fields due to their excellent thermal, mechanical, and biodegradable properties. Aerospace, automotive, packaging, military, construction, naval, sports, medical, and building block sectors represent significant applications of NBCs for obtaining superior performances, as shown in Figure 11 [128,180]. Bio-based composites provide lighter weight body parts for cars and airplanes, along with protection against heat and any external impacts. The tremendous interest and research studies on this sector are also gradually reducing the processing costs. The Toyota motor company proposed an eco-friendly bio-based car concept through designing polyester reinforced with hemp for lighter weight seats, body panels, carpets, and different interior parts [181]. NBCs also have very good potentiality for sustainable manufacturing through using green materials instead of traditional petroleum-based composites [182]. Electronics and mobile handsets have also drawn attention for implementing the green concept through reducing the harmful ingredients by replacing them with natural fiber-based products [179,183]. NBCs are considered as prominent safe and harmless materials for the environment, as they are manufactured from bio-based materials, so degradation occurs naturally, with CO_2_ and H_2_O as biproducts. The recycling of NBC is simple as the physical characteristics of nanofillers are not affected during the processing due to having a very good thermal stability. Besides, the low loading of nanofillers does not significantly increase the density of NBCs for the high aspect ratio, which provides a very high potentiality of using NBCs. Nanocellulose is gaining popularity in the biomedical industry for its use in scaffolds in tissue engineering, bone reconstruction, systems for drug release, the replacement of skin due to burning, and wound dressings [179]. 

## 9. Marketing Aspects of NBCs

The motives of prices and market potentiality are most important for obtaining adoption industrially, in comparison to the technical feasibility. Nanotechnologies have been estimated to have a market volume of $3 trillion throughout the world, with an employment value of six million workers [19]. The use of sustainable polymeric NBCs with low prices is very much in demand compared to conventional composites with satisfactory performance characteristics. Therefore, researchers and manufacturers are getting involved in finding more potential and novel routes of NBCs for consumers. Biofiber-based nanocomposites are now replacing petroleum-based fibers to produce NBCs, which is reducing the risk of greenhouse gas emissions and air pollution. On the other hand, the natural fiber-based BC production strategy could also generate huge employment for rural peoples to cultivate fibers [184]. Several studies have conducted a life cycle assessment (LCA) of BCs on the basis of environmental sustainability and reported a convenient biodegradability, waste recycling, renewability, a lighter weight, and a low fiber material cost [184,185,186]. However, the cost of some biopolymers, such as PLA and poly (ethylene glycol), is still a little higher, which is why the market success is facing challenges [187]. NBCs could receive more attentions from consumers if the cost could be minimized and convenient functionalities could be demonstrated (flame retardancy, water absorption, antibacterial characteristics, water repellency, energy absorption, moisture absorption, moisture content, and so on).

## 10. Conclusions

NBCs and BCs have shown a diverse innovative aptitude in the last few decades for noteworthy mechanical, thermal, and electrical characteristics and feasibility. Therefore, NBCs could meet the constantly increasing demand with advanced functionalities through enhancing performances in different sectors (aeronautical, automotive, construction, and so on). In the past few decades, fiber or particle reinforcements in composites have brought about some wonderful benchmarks that are making them popular among both manufacturers and consumers.

The classification of natural biofibers and characteristics of different nanoparticles have been studied to investigate and understand the potentiality and further improvement areas of NBCs. Natural fiber-based composites have an extreme potential to functionalize (waterproof, fire retardant, antibacterial property, UV-protection capability, insulation property, self-cleaning performances, and so on) with different nanoparticles (TiO_2_, rGO, SiO_2_, ZnO, and carbon nanotube), which could dominate research and application areas in the near future.

A huge array of different kinds of biofibers are available in nature, which could be easily fabricated as fiber reinforcing materials. Therefore, NBCs become biodegradable through ensuring sustainable technology in numerous applications.

NBCs are developed for specific purposes with different materials, but the qualities depend on the manufacturing processes, matrix properties, and types of nanomaterials and biofibers used. In general, manufacturing methods are designed based on the materials used. Therefore, the different physical parameters (tensile strength, melting point, and stiffness) of the materials should be considered before the selection of ingredients to make NBCs.

For certain applications, NBCs could replace traditional solo materials considering appropriate applications. The reduction in weight, strength, and stiffness exhibited by NBCs is magnificent, offering a versatile area for potential usage in transportation, construction, or electronics.

Furthermore, research studies are needed to divulge more scope to use NBC materials through varying the methods, technology, ingredients, or raw materials.

## Figures and Tables

**Table 1:** A brief summary on the merits and demerits of biofiber-reinforced biocomposites (BCs) and NBCs over traditional petroleum-based composites [11].

Number	Merits	Demerits
(a)	Comparatively lighter	Higher moisture absorption
(b)	Low cost	Low impact strength
(c)	Biodegradability	Poor flame retardancy
(d)	Renewability	Not suitable with a higher processing temperature
(e)	Better insulation and thermal performances	Poor resistance to microbial attack
(f)	Nontoxicity	Variation in quality
(g)	Environment-friendly	Complex supply chain of natural fibers for geographic locations and availability
(h)	No irritations with physical contact	/
(i)	Low energy consumption	/
(j)	Best alternatives for replacing synthetic fibers	/

**Table 2:** Chemical compositions of different natural fibers [11,38,39,40,41].

Fibers	Cellulose (%)	Hemi- cellulose (%)	Lignin (%)	Pectin (%)	Waxes (%)	Moisture Content/ Extractive (%)	Ash (%)
Cotton	89	4	0.75	6	0.6	--	--
Jute	45 to 71.5	13.6 to 21	12 to 26	0.2	--	12	0.5 to 2.0
Hemp	57 to 77	14 to 22.4	3.7 to 13	0.9	--	9	0.8
Flax	71	18.6 to 20.6	2.2	2.3	1.7	8 to 12	5 to 10
Coir	32 to 43	0.15 to 0.25	40 to 45	3 to 4	--	8	
Sisal	47 to 77	10 to 24	7 to 11	10	--	11	0.6 to 1.0
Kenaf	53.5	21	17	2	--	--	2 to 5
Sugarcane Bagasse	32 to 34	19 to 24	25 to 32	--	--	6 to 12	2 to 6
Bamboo	73.83	12.49	10.15	0.37	--	3.16-8.9	--
Ramie	68.6 to 91	5 to 16.7	0.6 to 0.7	1.9	--	9	--

**Table 3:** Typical properties of some selected natural fibers [11,34,53,54,55,56,57].

Fibers	Elongation (%)	Density (g/cm^3^)	Young’s Modulus (GPa)	Tensile Strength (MPa)	Decomposition Temperature (°C)
Cotton	3 to 10	1.5 to 1.6	5.5 to 12.6	287 to 597	232
Jute	1.5 to 1.8	1.3 to 1.46	10 to 30	393 to 800	215
Hemp	1.6	1.48	70	550 to 900	215
Flax	1.2 to 3.2	1.4 to 1.5	27.6 to 80	345 to 1500	220
Coir	15 to 30	1.2	4 to 6	175 to 220	285 to 465
Sisal	2 to 14	1.33 to 1.5	9 to 38	400 to 700	205 to 220
Kenaf	1.6 to 4.3	0.6 to 1.5	11 to 60	223 to 1191	229
Sugarcane Bagasse	6.3 to 7.9	1.1 to 1.6	5.1 to 6.2	170 to 350	232
Bamboo	1.9 to 3.2	1.2 to 1.5	27 to 40	500 to 575	214
Ramie	2.3 to 3.8	1.5	44 to 128	220 to 938	240

**Table 4:** Application, manufacturing method, and matrix materials of some potential natural fibers.

Reinforcing Fibers	Polymeric Matrix	Manufacturing Method	Application	Ref.
Cotton	Polylactic acid (PLA), silane, and low-density polyethylene (LDPE)	Extrusion and injection molding	Building, automotive, furniture, and food packaging	[72,73,74,75]
Jute	Polyester and PP	Compression/injection molding and hand lay-up	Door panels, ropes, roofing, durable chairs, kitchen sinks, sanitary latrines (slab and rings), helmets, and chest guards	[76,77]
Hemp	Polyethylene (PE), polyurethane (PU), and PP	Compression molding and resin transfer molding (RTM)	Automotives and furniture	[78,79]
Flax	Epoxy, PLA, polyester, and PP	Vacuum infusion, RTM, and hand lay-up	Textile, automotive and structural	[80,81]
Coir	PE, PP, and epoxy resin	Extrusion and injection molding	Building boards, insulation boards, roofing sheets, and automotive structural components	[82,83]
Sisal	Polystyrene(PS), PP, and epoxy resin	Compression molding and hand lay-up	Body parts of automobiles and roofing sheets	[84,85]
Kenaf	Epoxy resin, PLA, and PP	Pultrusion and compression molding	Bearings, automotive parts, and tooling	[86,87]
Sugarcane Bagasse	HDPE and poly (vinyl chloride) (PVC)	Compression molding, injection molding, and extrusion	Interior of automotives (side panels, seat frames, and central consuls)	[88,89]
Bamboo	Epoxy resin and PLA	Compression molding	Hardware for electronics, furniture, and toys	[90,91]
Ramie	PLA, PP, and polyolefin	Injection molding through extrusion	Civil and bulletproof vests	[92,93]

**Table 5:** The melting temperature (*T*_m_) and glass transition temperature (*T*_g_) of some commonly used resins [11].

Resin	Melting Temperature (*T*_m_) in °C	Glass Transition Temperature (*T*_g_) in °C
PLA	150 to 162	58
PP	160 to 176	0.9 to 1.55
Nylon 6	22	40
Polyester	250 to 300	60
LDPE	105 to 116	120
HDPE	120 to 140	80
Epoxy	-	70 to 67
Starch	110 to 115	60
Polystyrene	-	110–135

## References

[1] Zagho M.M., Hussein E.A., Elzatahry A.A. (2018). Recent Overviews in Functional Polymer Composites for Biomedical Applications. Polymers.

[2] Sherif G., Chukov D.I., Tcherdyntsev V.V., Torokhov V. (2019). Effect of Formation Route on the Mechanical Properties of the Polyethersulfone Composites Reinforced with Glass Fibers. Polymers.

[3] Mustafa A., Bin Abdollah M.F., Shuhimi F.F., Ismail N., Amiruddin H., Umehara N. (2015). Selection and verification of kenaf fibres as an alternative friction material using Weighted Decision Matrix method. Mater. Des..

[4] Mashkour M., Ranjbar Y. (2018). Superparamagnetic Fe_3_O_4_@ wood flour/polypropylene nanocomposites: Physical and mechanical properties. Ind. Crop. Prod..

[5] Sun G., Liang R., Lu Z., Shi T., Geng P., Li Z. (2017). Remarkable mechanical enhancement achieved by interfacial strengthening of organic/inorganic/fiber composites. Constr. Build. Mater..

[6] Sun Z., Mingming W. (2019). Effects of sol-gel modification on the interfacial and mechanical properties of sisal fiber reinforced polypropylene composites. Ind. Crop. Prod..

[7] Foruzanmehr M., Boulos L., Vuillaume P.Y., Elkoun S., Robert M. (2017). The Effect of cellulose oxidation on interfacial bonding of nano-TiO_2_ coating to flax fibers. Cellulose.

[8] Narayan R. (2009). Biodegradability. Bioplastics Magazine.

[9] Rahman R., Putra S.Z.F.S. (2019). Tensile properties of natural and synthetic fiber-reinforced polymer composites. Mechanical and Physical Testing of Biocomposites, Fibre-Reinforced Composites and Hybrid Composites.

[10] Gurunathan T., Mohanty S., Nayak S.K. (2015). A review of the recent developments in biocomposites based on natural fibres and their application perspectives. Compos. Part A Appl. Sci. Man..

[11] Gholampour A., Ozbakkaloglu T. (2019). A review of natural fiber composites: Properties, modification and processing techniques, characterization, applications. J. Mater. Sci..

[12] Davoodi M., Sapuan S., Ahmad D., Ali A., Khalina A., Jonoobi M., Sapuan S.M. (2010). Mechanical properties of hybrid kenaf/glass reinforced epoxy composite for passenger car bumper beam. Mater. Des..

[13] Tshai K.Y., Kong I. (2020). Advancement in flame retardancy of natural fibre reinforced composites with macro to nanoscale particulates additives. Interfaces in Particle and Fibre Reinforced Composites.

[14] Wu H., Fahy W., Kim S., Kim H., Zhao N., Pilato L., Kafi A., Bateman S., Koo J. (2020). Recent developments in polymers/polymer nanocomposites for additive manufacturing. Prog. Mater. Sci..

[15] Thakur V.K., Thakur M.K. (2014). Processing and characterization of natural cellulose fibers/thermoset polymer composites. Carbohydr. Polym..

[16] Al-Oqla F.M., Sapuan S., Anwer T., Jawaid M., Hoque E., Sapuan S.M. (2015). Natural fiber reinforced conductive polymer composites as functional materials: A review. Synth. Met..

[17] Kord B., Ghalehno M.D., Movahedi F. (2019). Effect of Surface Functionalization of SiO2 Nanoparticles on the Dynamic Mechanical, Thermal and Fire Properties of Wheat Straw/LDPE Composites. J. Polym. Environ..

[18] Mousa M., Dong Y. (2020). A critical role of interphase properties and features on mechanical properties of poly(vinyl alcohol) (PVA) bionanocomposites. Interfaces in Particle and Fibre Reinforced Composites.

[19] Saba N., Paridah M.T., Jawaid M. (2014). A Review on Potentiality of Nano Filler/Natural Fiber Filled Polymer Hybrid Composites. Polymers.

[20] Islam S., Ahmad M.B., Hasan M., Aziz S.A., Jawaid M., Haafiz M.K.M., Zakaria S.A.H. (2015). Natural Fiber-Reinforced Hybrid Polymer Nanocomposites: Effect of Fiber Mixing and Nanoclay on Physical, Mechanical, and Biodegradable Properties. Bioresources.

[21] Ching Y.C., Rahman A., Ching K.Y., Sukiman N.L., Cheng H.C. (2015). Preparation and Characterization of Polyvinyl Alcohol-Based Composite Reinforced with Nanocellulose and Nanosilica. Bioresources.

[22] Sharma A., Bhojak V., Kukshal V., Biswas S.K., Patnaik A., Patnaik T.K. (2019). Mechanical and Erosion Characteristics of Natural Fiber Reinforced Polymer Composite: Effect of Filler Size. Automotive Tribology. Energy, Environment, and Sustainability.

[23] Arjmandi R., Hassan A., Zakaria Z. (2017). Polylactic Acid Green Nanocomposites for Automotive Applications. Global Warming.

[24] Kong J., Tan B.H., Lu X., Li Z., He C. (2020). Hybrid POSS Nanocomposites. Silicon Containing Hybrid Copolymers.

[25] Chen H., Cui Y., Liu X., Zhang M., Hao S., Yin Y. (2019). Study on depositing SiO 2 nanoparticles on the surface of jute fiber via hydrothermal method and its reinforced polypropylene composites. J. Vinyl Addit. Technol..

[26] Spinella A., Bondioli F., Nasillo G., Renda V., Caponetti E., Messori M., Morselli D. (2017). Organic-inorganic nanocomposites prepared by reactive suspension method: Investigation on filler/matrix interactions and their effect on the nanoparticles dispersion. Colloid Polym. Sci..

[27] Bansal S.A., Singh A.P., Kumar A., Kumar S., Kumar N., Goswamy J.K. (2017). Improved mechanical performance of bisphenol-A graphene-oxide nano-composites. J. Compos. Mater..

[28] Genix A.-C., Oberdisse J. (2018). Nanoparticle self-assembly: From interactions in suspension to polymer nanocomposites. Soft Matter.

[29] Jeyapragash R., Srinivasan V., Sathiyamurthy S. (2020). Mechanical properties of natural fiber/particulate reinforced epoxy composites—A review of the literature. Mater. Today Proc..

[30] Balla V.K., Kate K.H., Satyavolu J., Singh P., Tadimeti J.G.D. (2019). Additive manufacturing of natural fiber reinforced polymer composites: Processing and prospects. Compos. Part B Eng..

[31] Amiandamhen S., Meincken M., Tyhoda L. (2020). Natural Fibre Modification and Its Influence on Fibre-matrix Interfacial Properties in Biocomposite Materials. Fiber. Polym..

[32] Markó G., Halász K., Alpár T. Natural fibre reinforced PLA composite. Proceedings of the International Conference on Bio-Friendly Polymers and Polymer Additives: From Scientific Aspects to Processing and Applications.

[33] Omrani E., Menezes P.L., Rohatgi P.K. (2016). State of the art on tribological behavior of polymer matrix composites reinforced with natural fibers in the green materials world. Eng. Sci. Technol. Int. J..

[34] Rajak D.K., Pagar D.D., Menezes P.L., Linul E. (2019). Fiber-Reinforced Polymer Composites: Manufacturing, Properties, and Applications. Polymers.

[35] Kamaraj M., Dodson E.A., Datta S. (2019). Effect of graphene on the properties of flax fabric reinforced epoxy composites. Adv. Compos. Mater..

[36] Shea A., Lawrence M., Walker P., Walker P. (2012). Hygrothermal performance of an experimental hemp–lime building. Constr. Build. Mater..

[37] Hussain A., Calabria-Holley J., Lawrence M., Ansell M.P., Jiang Y., Schorr D., Blanchet P. (2019). Development of novel building composites based on hemp and multi-functional silica matrix. Compos. Part B Eng..

[38] Bajpai P., Marinakis K.K., Fisher M.W. (2018). Biermann’s Handbook of Pulp and Paper: Paper and Board Making.

[39] Azeez M.A., Orege J.I. (2018). Bamboo, Its Chemical Modification and Products. Bamboo Curr. Fut. Prosp..

[40] Li X., Tabil L., Panigrahi S. (2007). Chemical Treatments of Natural Fiber for Use in Natural Fiber-Reinforced Composites: A Review. J. Polym. Environ..

[41] Komuraiah A., Kumar N.S., Prasad B.D. (2014). Chemical Composition of Natural Fibers and its Influence on their Mechanical Properties. Mech. Compos. Mater..

[42] Ayrilmis N., Jarusombuti S., Fueangvivat V., Bauchongkol P., White R.H. (2011). Coir fiber reinforced polypropylene composite panel for automotive interior applications. Fibers Polym..

[43] Muzyczek M. (2020). The use of flax and hemp for textile applications. Handbook of Natural Fibres.

[44] Zhang Y., Li Y., Ma H., Yu T. (2013). Tensile and interfacial properties of unidirectional flax/glass fiber reinforced hybrid composites. Compos. Sci. Technol..

[45] Yan L., Chouw N., Jayaraman K. (2014). Flax fibre and its composites—A review. Compos. Part B Eng..

[46] Battegazzore D., Frache A., Abt T., Maspoch M.L. (2018). Epoxy coupling agent for PLA and PHB copolymer-based cotton fabric bio-composites. Compos. Part B Eng..

[47] Holt G., Chow P., Wanjura J., Pelletier M., Wedegaertner T. (2014). Evaluation of thermal treatments to improve physical and mechanical properties of bio-composites made from cotton byproducts and other agricultural fibers. Ind. Crop. Prod..

[48] Shibata M., Teramoto N., Nakamura T., Saitoh Y. (2013). All-cellulose and all-wood composites by partial dissolution of cotton fabric and wood in ionic liquid. Carbohydr. Polym..

[49] Shen M., Wang L., Long J.-J. (2015). Biodegumming of ramie fiber with pectinases enhanced by oxygen plasma. J. Clean. Prod..

[50] Roy S., Lutfar L. (2012). Bast fibres: Ramie. Handbook of Natural Fibres.

[51] Mahmud S., Pervez N., Hasan K.M.F., Abu Taher M., Liu H., Mahmud S. (2019). In situ synthesis of green AgNPs on ramie fabric with functional and catalytic properties. Emerg. Mater. Res..

[52] Yu T., Ren J., Li S., Yuan H., Li Y. (2010). Effect of fiber surface-treatments on the properties of poly (lactic acid)/ramie composites. Compos. Part A Appl. Sci. Manuf..

[53] Verma D., Senal I., Verma D., Fortunati E., Jain S., Zhang X. (2019). Natural fiber-reinforced polymer composites: Feasibiliy study for sustainable automotive industries. Biomass, Biopolymer-Based Materials, and Bioenergy.

[54] Menezes P.L., Rohatgi P., Lovell M.R. (2012). Studies on the Tribological Behavior of Natural Fiber Reinforced Polymer Composite.

[55] Razali N., Sapuan S.M., Jawaid M., Ishak M.R., Lazim Y. (2015). A Study on Chemical Composition, Physical, Tensile, Morphological, and Thermal Properties of Roselle Fibre: Effect of Fibre Maturity. Bioresources.

[56] Pereira J.F., Ferreira D., Bessa J., Matos J., Cunha F., Araújo I., Silva L.F., Pinho E., Fangueiro R. (2019). Mechanical performance of thermoplastic olefin composites reinforced with coir and sisal natural fibers: Influence of surface pretreatment. Polym. Compos..

[57] Essabir H., Bensalah M., Rodrigue D., Bouhfid R., Qaiss A.E.K. (2016). Structural, mechanical and thermal properties of bio-based hybrid composites from waste coir residues: Fibers and shell particles. Mech. Mater..

[58] Zhu Z., Hao M., Zhang N. (2018). Influence of contents of chemical compositions on the mechanical property of sisal fibers and sisal fibers reinforced PLA composites. J. Nat. Fibers.

[59] Hempalaya The Difference between Hemp and Linen Fibers. https://hempalaya.com/blogs/news/der-unterschied-zwischen-hanf-und-leinen-fasern.

[60] Sunstrands The Basics of Kenaf Fiber and Hurd. https://www.sunstrands.com/2019/the-uses-of-kenaf-fiber/.

[61] Swadesh Indira Gandhi Krishi Vishvavidyalaya (IGKV) Achieves a Breakthrough in Getting Linen Yarn Using the Flax Plant. https://www.unnatisilks.com/blog/indira-gandhi-krishi-vishvavidyalaya-igkv-achieves-a-breakthrough-in-getting-linen-yarn-using-the-flax-plant/.

[62] Textiles School Sampling from Cotton Bales. https://www.textileschool.com/164/cotton-fibers-and-its-properties.

[63] Fatema Jute Fibers Raw Jute. http://fatemajutefibers.com.

[64] Always Fresh Why We Use Coconut Coir Not Soil. http://www.alwaysfresh2u.com/uncategorized/use-coconut-coir-not-soil.

[65] Tintsaba The Sesal Plant. https://www.tintsaba.com/sisal.

[66] Leiden T. Ramie. https://trc-leiden.nl/trc-needles/materials/fibres/ramie.

[67] Fan R., Ma W., Liu S., Huang Q. (2019). Integrated analysis of four new sequenced fern Chloroplast Genomes: Genome structure and Comparative Analysis. Plant Physiol. Morphol..

[68] Dreamstime Sugarcane Juice Gabasse. https://www.dreamstime.com/photos-images/sugarcane-juice-bagasse.html.

[69] Cisneros-López E., Pérez-Fonseca A.A., Fuentes-Talavera F., Anzaldo J., González-Núñez R., Rodrigue D., Robledo-Ortíz J.R. (2016). Rotomolded polyethylene-agave fiber composites: Effect of fiber surface treatment on the mechanical properties. Polym. Eng. Sci..

[70] Langhorst A.E., Burkholder J., Long J., Thomas R., Kiziltas A., Mielewski D. (2017). Blue-Agave Fiber-Reinforced Polypropylene Composites for Automotive Applications. Bioresources.

[71] Annandarajah C., Li P., Michel M., Chen Y., Jamshidi R., Kiziltas A., Hoch R., Grewell D., Montazami R. (2018). Study of Agave Fiber-Reinforced Biocomposite Films. Materials.

[72] Mahdi E., Dean A. (2020). The Effect of Filler Content on the Tensile Behavior of Polypropylene/Cotton Fiber and poly (vinyl chloride)/Cotton Fiber Composites. Materials.

[73] Ng W., Johar M., Israr H., Wong K. (2020). A review on the interfacial characteristics of natural fibre reinforced polymer composites. Interfaces in Particle and Fibre Reinforced Composites.

[74] Farahbakhsh N., Shahbeigi-Roodposhti P., Sadeghifar H., Venditti R.A., Jur J.S. (2017). Effect of isolation method on reinforcing capability of recycled cotton nanomaterials in thermoplastic polymers. J. Mater. Sci..

[75] Battegazzore D., Abt T., Maspoch M.L., Frache A. (2019). Multilayer cotton fabric bio-composites based on PLA and PHB copolymer for industrial load carrying applications. Compos. Part B Eng..

[76] Ashraf M.A., Zwawi M., Mehran M.T., Kanthasamy R., Bahadar A. (2019). Jute Based Bio and Hybrid Composites and Their Applications. Fibers.

[77] Dinesh S., Kumaran P., Mohanamurugan S., Vijay R., Singaravelu D.L., Vinod A., Sanjay M., Siengchin S., Bhat K.S. (2019). Influence of wood dust fillers on the mechanical, thermal, water absorption and biodegradation characteristics of jute fiber epoxy composites. J. Polym. Res..

[78] Tanasă F., Zănoagă M., Teacă C., Nechifor M., Shahzad A. (2019). Modified hemp fibers intended for fiber-reinforced polymer composites used in structural applications—A review. I. Methods of modification. Polym. Compos..

[79] Del Borrello M., Mele M., Campana G., Secchi M. (2020). Manufacturing and characterization of hemp-reinforced epoxy composites. Polym. Compos..

[80] Aliotta L., Gigante V., Coltelli M.-B., Cinelli P., Lazzeri A., Seggiani M. (2019). Thermo-Mechanical Properties of PLA/Short Flax Fiber Biocomposites. Appl. Sci..

[81] Zhang H., Liu D., Huang T., Hu Q., Lammer H. (2020). Three-Dimensional Printing of Continuous Flax Fiber-Reinforced Thermoplastic Composites by Five-Axis Machine. Materials.

[82] Munde Y.S., Ingle R.B., Siva I. (2018). Investigation to appraise the vibration and damping characteristics of coir fibre reinforced polypropylene composites. Adv. Mater. Process. Technol..

[83] Mishra S., Nayak C., Sharma M.K., Dwivedi U.K. (2020). Influence of Coir Fiber Geometry on Mechanical Properties of SiC Filled Epoxy Composites. Silicon.

[84] Saxena M., Pappu A., Haque R., Sharma A. (2011). Sisal Fiber Based Polymer Composites and Their Applications. Cellulose Fibers: Bio- and Nano-Polymer Composites.

[85] Chaitanya S., Singh I., Song J.-I. (2019). Recyclability analysis of PLA/Sisal fiber biocomposites. Compos. Part B Eng..

[86] Manral A., Bajpai P.K. (2019). Static and dynamic mechanical analysis of geometrically different kenaf/PLA green composite laminates. Polym. Compos..

[87] Asumani O., Paskaramoorthy R. (2020). Fatigue and impact strengths of kenaf fibre reinforced polypropylene composites: Effects of fibre treatments. Adv. Compos. Mater..

[88] Sitticharoen W., Chainawakul A., Sangkas T., Kuntham Y. (2016). Rheological and Mechanical Properties of Silica-Based Bagasse-Fiber-Ash-Reinforced Recycled HDPE Composites. Mech. Compos. Mater..

[89] Wirawan R., Sapuan S. (2018). Sugarcane Bagasse-Filled Poly(Vinyl Chloride) Composites. Natural Fibre Reinforced Vinyl Ester and Vinyl Polymer Composites.

[90] Das M. (2016). Bamboo Fiber-Based Polymer Composites. Polymer-Engineered Nanostructures for Advanced Energy Applications.

[91] Lokesh P., Kumari T.S., Gopi R., Loganathan G.B. (2020). A study on mechanical properties of bamboo fiber reinforced polymer composite. Mater. Today Proc..

[92] Choi H.Y., Lee J.-S. (2012). Effects of surface treatment of ramie fibers in a ramie/poly(lactic acid) composite. Fibers Polym..

[93] Djafar Z., Renreng I., Jannah M. (2020). Tensile and Bending Strength Analysis of Ramie Fiber and Woven Ramie Reinforced Epoxy Composite. J. Nat. Fibers.

[94] Chandar J.V., Shanmugan S., Murugan P., Mutharasu D., Sudesh K. (2016). Structural Analysis of ZnO Nanoparticles Reinforced P(3HB-co-15 mol% 3HHx) Bioplastic Composite. J. Polym. Environ..

[95] Karakoti A., Biswas S., Aseer J.R., Sindhu N., Sanjay M. (2018). Characterization of microfiber isolated from Hibiscus sabdariffa var. altissima fiber by steam explosion. J. Nat. Fibers.

[96] Phanthong P., Karnjanakom S., Reubroycharoen P., Hao X., Abudula A., Guan G. (2017). A facile one-step way for extraction of nanocellulose with high yield by ball milling with ionic liquid. Cellulose..

[97] Souza S., Ferreira F., Cherian B., Silva V., Manzato L., Pinheiro I. (2020). Processing of nanocellulose-based composites. Fiber-Reinforced Nanocomposites: Fundamentals and Applications.

[98] Miyashiro D., Hamano R., Umemura K. (2020). A Review of Applications Using Mixed Materials of Cellulose, Nanocellulose and Carbon Nanotubes. Nanomaterials.

[99] Martin-Martinez F.J. (2018). Designing nanocellulose materials from the molecular scale. Proc. Natl. Acad. Sci. USA.

[100] Dixit S., Goel R., Dubey A., Shivhare P.R., Bhalavi T. (2017). Natural Fibre Reinforced Polymer Composite Materials - A Review. Polym. Renew. Resour..

[101] Nassar A., Nassar E. (2020). Effect of fiber orientation on the mechanical properties of multi layers laminate nanocomposites. Heliyon.

[102] Crosky A., Soatthiyanon N., Ruys D., Meatherall S., Potter S. (2014). Thermoset matrix natural fibre-reinforced composites. Natural Fibre Composites.

[103] Mohammed L., Ansari M.N.M., Pua G., Jawaid M., Islam S. (2015). A Review on Natural Fiber Reinforced Polymer Composite and Its Applications. Int. J. Polym. Sci..

[104] Ghosh A.K., Dwivedi M. (2019). Processability in Open Mould Processing of Polymeric Composites. Processability of Polymeric Composites.

[105] Hosseini S.B. (2020). Natural fiber polymer nanocomposites. Fiber-Reinforced Nanocomposites: Fundamentals and Applications.

[106] Faruk O., Bledzki A.K., Fink H.-P., Sain M. (2012). Biocomposites reinforced with natural fibers: 2000–2010. Prog. Polym. Sci..

[107] Alharbi M.A.H., Hirai S., Tuan H.A., Akioka S., Shoji W. (2020). Effects of chemical composition, mild alkaline pretreatment and particle size on mechanical, thermal, and structural properties of binderless lignocellulosic biopolymers prepared by hot-pressing raw microfibrillated Phoenix dactylifera and Cocos nucifera fibers and leaves. Polym. Test..

[108] Verma A., Parashar A., Jain N., Singh V.K., Rangappa S.M., Siengchin S. (2020). Surface Modification Techniques for the Preparation of Different Novel Biofibers for Composites. Biofibers and Biopolymers for Biocomposites.

[109] Franco-Marquès E., Méndez J.A., Pèlach M., Àngels, Vilaseca F., Bayer J., Mutjé P. (2011). Influence of coupling agents in the preparation of polypropylene composites reinforced with recycled fibers. Chem. Eng. J..

[110] Xie Y., Hill C.A., Xiao Z., Militz H., Mai C. (2010). Silane coupling agents used for natural fiber/polymer composites: A review. Compos. Part A Appl. Sci. Manuf..

[111] Kabir M., Wang H., Lau K., Cardona F. (2012). Chemical treatments on plant-based natural fibre reinforced polymer composites: An overview. Composites Part B: Engineering..

[112] Campilho R.D.S.G. (2015). Natural Fiber Composites.

[113] DiTommaso G., Gaff M., Kačík F., Sikora A., Sethy A., Corleto R., Razaei F., Kaplan L., Kubš J., Das S. (2020). Interaction of technical and technological factors on qualitative and energy/ecological/economic indicators in the production and processing of thermally modified merbau wood. J. Clean. Prod..

[114] AL-Oqla F.M., Omari M.A. (2017). Sustainable biocomposites: Challenges, potential and barriers for development. Green biocomposites.

[115] Navaneethakrishnan G., Karthikeyan T., Saravanan S., Selvam V., Parkunam N., Sathishkumar G., Jayakrishnan S. (2020). Structural analysis of natural fiber reinforced polymer matrix composite. Mater. Today: Proc..

[116] Jaafar J., Siregar J.P., Tezara C., Hamdan M.H.M., Rihayat T. (2019). A review of important considerations in the compression molding process of short natural fiber composites. Int. J. Adv. Manuf. Technol..

[117] Sarikaya E., Çallioğlu H., Demirel H. (2019). Production of epoxy composites reinforced by different natural fibers and their mechanical properties. Compos. Part B Eng..

[118] Avella M., Buzarovska A., Errico M., Gentile G., Grozdanov A. (2009). Eco-Challenges of Bio-Based Polymer Composites. Materials.

[119] Crawford B., Pakpour S., Kazemian N., Klironomos J., Stoeffler K., Rho D., Denault J., Milani A.S. (2017). Effect of Fungal Deterioration on Physical and Mechanical Properties of Hemp and Flax Natural Fiber Composites. Materials.

[120] Chieng B.W., Ibrahim N.A., Then Y.Y., Loo Y.Y. (2014). Epoxidized Vegetable Oils Plasticized Poly(lactic acid) Biocomposites: Mechanical, Thermal and Morphology Properties. Moleluces.

[121] Wang Z., Kang H., Zhang J., Zhang S., Li J. (2017). Improvement of Interfacial Adhesion by Bio-Inspired Catechol-Functionalized Soy Protein with Versatile Reactivity: Preparation of Fully Utilizable Soy-Based Film. Polymers.

[122] Liu W., Fei M.-E., Ban Y., Jia A., Qiu R. (2017). Preparation and Evaluation of Green Composites from Microcrystalline Cellulose and a Soybean-Oil Derivative. Polymers.

[123] Alpár T., Markó G., Koroknai L. (2017). Natural Fiber Reinforced PLA Composites: Effect of Shape of Fiber Elements on Properties of Composites. Handbook of Composites from Renewable Materials.

[124] Getme A.S., Patel B. (2020). A Review: Bio-fiber’s as reinforcement in composites of polylactic acid (PLA). Mater. Today Proc..

[125] Miao C., Hamad W.Y. (2013). Cellulose reinforced polymer composites and nanocomposites: A critical review. Cellulose.

[126] Cótica L.F., Garcia A., Polli A.D., Bini R.D., De Chaves T., Junior V.A.D.O., Pamphile J.A. (2018). Nanobiocomposites: Synthesis and Environmental Applications. Fungal Nanobionics: Principles and Applications.

[127] Iozzino V., Haroutioun A., Fabrice L., Vincent V., Pantani R. (2018). Poly(Lactic Acid)-Based Nanobiocomposites with Modulated Degradation Rates. Materials.

[128] Kausar A. (2020). Progress in green nanocomposites for high-performance applications. Mater. Res. Innov..

[129] Adamu M., Rahman M.R., Hamdan S. (2020). Bamboo Nanocomposite: Impact of Poly (Ethylene-alt-Maleic Anhydride) and Nanoclay on Physicochemical, Mechanical, and Thermal Properties. BioResources.

[130] Saba N., Paridah M., Abdan K., Ibrahim N. (2016). Physical, structural and thermomechanical properties of oil palm nano filler/kenaf/epoxy hybrid nanocomposites. Mater. Chem. Phys..

[131] Sinha R.K., Sridhar K., Purohit R., Malviya R.K. (2020). Effect of nano SiO2 on properties of natural fiber reinforced epoxy hybrid composite: A review. Mater. Today: Proc..

[132] Lazzara G., Cavallaro G., Panchal A., Fakhrullin R., Stavitskaya A., Vinokurov V.A., Lvov Y.M. (2018). An assembly of organic-inorganic composites using halloysite clay nanotubes. Curr. Opin. Colloid Interface Sci..

[133] Blanco I. (2018). The Rediscovery of POSS: A Molecule Rather than a Filler. Polymers.

[134] Mohajerani A., Burnett L., Smith J.V., Kurmus H., Milas J., Arulrajah A., Horpibulsuk S., Kadir A., Kadir A.A. (2019). Nanoparticles in Construction Materials and Other Applications, and Implications of Nanoparticle Use. Materials.

[135] Marquis D.M., Guillaume E., Chivas-Joly C. (2011). Properties of Nanofillers in Polymer. Nanocomposites and Polymers with Analytical Methods.

[136] Nguyen T.A., Han B., Sharma S., Longbiao L., Bhat K.S. (2020). Fiber-reinforced nanocomposites: An introduction. Fiber-Reinforced Nanocomposites: Fundamentals and Applications.

[137] Sharma R.K., Sharma S., Dutta S., Zboril R., Gawande M.B. (2015). Silica-nanosphere-based organic–inorganic hybrid nanomaterials: Synthesis, functionalization and applications in catalysis. Green Chem..

[138] Yang Y., Zhang C., Lai C., Zeng G., Huang D., Cheng M., Wang J., Chen F., Zhou C., Xiong W. (2018). BiOX (X = Cl, Br, I) photocatalytic nanomaterials: Applications for fuels and environmental management. Adv. Colloid Interface Sci..

[139] Rajini N., Jappes J.W., Siva I., Rajulu A.V., Rajakarunakaran S. (2016). Fire and thermal resistance properties of chemically treated ligno-cellulosic coconut fabric–reinforced polymer eco-nanocomposites. J. Ind. Text..

[140] Ramu P., Kumar C.J., Palanikumar K. (2019). Mechanical Characteristics and Terminological Behavior Study on Natural Fiber Nano reinforced Polymer Composite—A Review. Mater. Today Proc..

[141] Srivastava V.K., Gries T., Veit D., Quadflieg T., Mohr B., Kolloch M. (2017). Effect of nanomaterial on mode I and mode II interlaminar fracture toughness of woven carbon fabric reinforced polymer composites. Eng. Fract. Mech..

[142] Prasad V., Sekar K., Varghese S., Joseph M. (2019). Enhancing Mode I and Mode II interlaminar fracture toughness of flax fibre reinforced epoxy composites with nano TiO_2_. Compos. Part A Appl. Sci. Manuf..

[143] Jujjavarapu S.E., Poluri K.M. (2020). Green Polymeric Nanocomposites.

[144] Pinto D., Bernardo L., Amaro A., Lopes S.M.R. (2015). Mechanical properties of epoxy nanocomposites using titanium dioxide as reinforcement—A review. Constr. Build. Mater..

[145] Catauro M., Barrino F., Blanco I., Piccolella S., Pacifico S. (2020). Use of the Sol–Gel Method for the Preparation of Coatings of Titanium Substrates with Hydroxyapatite for Biomedical Application. Coatings.

[146] Jiang Q., Pei X., Wu L., Li T.-T., Lin J.-H. (2018). UV resistance and water barrier properties of PP/PLA/MAH/TiO_2_ functional hybrid biocomposite films for packaging application. Adv. Polym. Technol..

[147] Brandrup J., Immergut E.H., Grulke E.A., Abe A., Bloch D.R. (1999). Polymer Handbook.

[148] Battegazzore D. (2011). Crystallization kinetics of poly(lactic acid)-talc composites. Express Polym. Lett..

[149] Nomai J., Suksut B., Schlarb A.K. (2015). Crystallization behavior of poly (lactic acid)/titanium dioxide nanocomposites. KMUTNB. Int. J. Appl. Sci. Technol..

[150] Prasad V., Joseph M., Sekar K. (2018). Investigation of mechanical, thermal and water absorption properties of flax fibre reinforced epoxy composite with nano TiO_2_ addition. Compos. Part A Appl. Sci. Manuf..

[151] Mousavi S.M., Hashemi S.A., Jahandideh S., Baseri S., Zarei M., Azadi S. (2017). Modification of Phenol Novolac Epoxy Resin and Unsaturated Polyester Using Sasobit and Silica Nanoparticles. Polym. Renew. Resour..

[152] Hashemi S.A., Mousavi S.M. (2016). Effect of bubble based degradation on the physical properties of Single Wall Carbon Nanotube/Epoxy Resin composite and new approach in bubbles reduction. Compos. Part A Appl. Sci. Manuf..

[153] Rahman M.R., Rahman M., Hamdan S., Lai J.C.H. (2016). Impact of Maleic Anhydride, Nanoclay, and Silica on Jute Fiber-reinforced Polyethylene Biocomposites. Bioresources.

[154] Chang H., Sun S.-Q., Huan C., Sun S. (2014). Silicon nanoparticles: Preparation, properties, and applications. Chin. Phys. B.

[155] Catauro M., Barrino F., Blanco I., Dal Poggetto G., Piccolella S., Crescente G., Pacifico S. (2020). Bioactivity of chlorogenic acid/SiO2/PEG composite synthesized via sol-gel. Mater. Today..

[156] Siengchin S., Dangtungee R. (2014). Polyethylene and polypropylene hybrid composites based on nano silicon dioxide and different flax structures. J. Thermoplast. Compos. Mater..

[157] Ma L., Zhou M., He C., Li S., Fan X., Nie C., Luo H., Qiu L., Cheng C. (2019). Graphene-based advanced nanoplatforms and biocomposites from environmentally friendly and biomimetic approaches. Green Chem..

[158] Kumar D., Babu G., Krishnan S. (2019). Study on mechanical & thermal properties of PCL blended graphene biocomposites. Polímeros.

[159] Idumah C.I., Hassan A. (2017). Hibiscus Cannabinus Fiber/PP based Nano-Biocomposites Reinforced with Graphene Nanoplatelets. J. Nat. Fibers.

[160] Zhang Y., Gong S., Zhang Q., Ming P., Wan S., Peng J., Jiang L., Cheng Q. (2016). Graphene-based artificial nacre nanocomposites. Chem. Soc. Rev..

[161] Gong S., Ni H., Jiang L., Cheng Q. (2017). Learning from nature: Constructing high performance graphene-based nanocomposites. Mater. Today.

[162] Razak Z., Sulong A.B., Muhamad N., Haron C.H.C., Radzi M.K.F.M., Ismail N.F., Tholibon D., Tharazi I. (2019). Effects of thermal cycling on physical and tensile properties of injection moulded kenaf/carbon nanotubes/polypropylene hybrid composites. Compos. Part B Eng..

[163] Fu S., Song P., Yang H., Jin Y., Lu F., Ye J., Wu Q. (2010). Effects of carbon nanotubes and its functionalization on the thermal and flammability properties of polypropylene/wood flour composites. J. Mater. Sci..

[164] Russo P., Vitiello L., Sbardella F., Santos J.I., Tirillò J., Bracciale M.P., Rivilla I., Sarasini F. (2020). Effect of Carbon Nanostructures and Fatty Acid Treatment on the Mechanical and Thermal Performances of Flax/Polypropylene Composites. Polymers.

[165] Montazeri A., Javadpour J., Khavandi A., Tcharkhtchi A., Mohajeri A. (2010). Mechanical properties of multi-walled carbon nanotube/epoxy composites. Mater. Des..

[166] Tzounis L., Debnath S., Rooj S., Fischer D., Mäder E., Das A., Stamm M., Heinrich G. (2014). High performance natural rubber composites with a hierarchical reinforcement structure of carbon nanotube modified natural fibers. Mater. Des..

[167] Li Y., Cai S., Huang X. (2017). Multi-scaled enhancement of damping property for carbon fiber reinforced composites. Compos. Sci. Technol..

[168] Turlybekuly A., Pogrebnjak A., Sukhodub L., Sukhodub L., Kistaubayeva A., Savitskaya I., Shokatayeva D., Bondar O., Shaimardanov Z., Plotnikov S. (2019). Synthesis, characterization, in vitro biocompatibility and antibacterial properties study of nanocomposite materials based on hydroxyapatite-biphasic ZnO micro- and nanoparticles embedded in Alginate matrix. Mater. Sci. Eng. C.

[169] Al Abdullah K., Awad S., Zaraket J., Salame C.-T. (2017). Synthesis of ZnO Nanopowders By Using Sol-Gel and Studying Their Structural and Electrical Properties at Different Temperature. Energy Procedia.

[170] Koronis G., Silva A. (2018). Green Composites for Automotive Applications.

[171] Less by Design (2015). CocoForm-a Sustainable Packaging Alternative. https://lessbydesign.org/2015/02/06/cocoform-a-sustainable-packaging-alternative.

[172] Architonic (2018). Biocomposites Experimental Pavilion, Stuttgart, Germany. https://www.architonic.com/en/project/biomat-group-at-itke-biocomposites-experimental-pavilion/20013658.

[173] Treehugger Cannabis in Your Car Doors, but Not to Smuggle It. https://www.treehugger.com/sustainable-agriculture/cannabis-your-car-doors-not-smuggle-it.html.

[174] Godavari Natural Fiber Biocomposite. https://www.somaiya.com/naturomer-%C2%AE-natural-fiber-biocomposite.

[175] Composites World Six Candidates Nominated for “Biocomposite of the Year 2019”. https://www.compositesworld.com/news/six-candidates-nominated-for-biocomposite-of-the-year-2019.

[176] Siti S., Abdul H., Wan W., Jawai M. (2013). Bamboo Based Biocomposites Material, Design and Applications. Materials Science—Advanced Topics.

[177] Reddy T.R.K., Kim H., Park J.-W. (2016). Renewable Biocomposite Properties and their Applications. Composites from Renewable and Sustainable Materials.

[178] John M.J., Thomas S. (2008). Biofibres and biocomposites. Carbohydr. Polym..

[179] Kalia S., Dufresne A., Cherian B.M., Kaith B., Avérous L., Njuguna J., Nassiopoulos E. (2011). Cellulose-Based Bio- and Nanocomposites: A Review. Int. J. Polym. Sci..

[180] Christy P.N., Basha S.K., Kumari V.S., Bashir A., Maaza M., Kaviyarasu K., Arasu M.V., Al-Dhabi N.A., Ignacimuthu S. (2020). Biopolymeric nanocomposite scaffolds for bone tissue engineering applications—A review. J. Drug Deliv. Sci. Technol..

[181] Akampumuza O., Wambua P.M., Ahmed A., Li W., Qin X.-H. (2016). Review of the applications of biocomposites in the automotive industry. Polym. Compos..

[182] Arora B., Bhatia R., Attri P. (2018). Bionanocomposites: Green materials for a sustainable future. New Polymer Nanocomposites for Environmental Remediation.

[183] Dicker M., Duckworth P.F., Baker A.B., François G., Hazzard M.K., Weaver P.M. (2014). Green composites: A review of material attributes and complementary applications. Compos. Part A Appl. Sci. Manuf..

[184] Malviya R.K., Singh R.K., Purohit R., Sinha R. (2020). Natural fibre reinforced composite materials: Environmentally better life cycle assessment—A case study. Mater. Today Proc..

[185] Ita-Nagy D., Vázquez-Rowe I., Kahhat R., Quispe I., Chinga-Carrasco G., Clauser N.M., Area M.C. (2020). Life cycle assessment of bagasse fiber reinforced biocomposites. Sci. Total. Environ..

[186] Sarkar S., Gulati K., Poluri K.M. (2020). Life Cycle Assessment and Future Perspectives of Green Polymeric Nanocomposites. Green Polymeric Nanocomposites.

[187] Rujnić-Sokele M., Pilipović A. (2017). Challenges and opportunities of biodegradable plastics: A mini review. Waste Manag. Res..

